# Newly Synthesized Telmisartan–Amino Acid Conjugates Exhibit Enhanced Cytotoxic Effects in Malignant Melanoma Cells

**DOI:** 10.3390/molecules31010125

**Published:** 2025-12-29

**Authors:** Dragana Vukadinović, Ana Damjanović, Miodrag Vuković, Olivera Čudina, Jelena Grahovac, Vladimir Dobričić

**Affiliations:** 1 Department of Pharmaceutical Chemistry, University of Belgrade—Faculty of Pharmacy, Vojvode Stepe 450, 11221 Belgrade, Serbia; ph.dragana@gmail.com (D.V.);; 2 Department for Pharmacy, Military Medical Academy, Crnotravska 17, 11000 Belgrade, Serbia; 3 Department of Experimental Oncology, Institute for Oncology and Radiology of Serbia, Pasterova 14, 11000 Belgrade, Serbia

**Keywords:** telmisartan–amino acid conjugates, anticancer effects, synthesis, melanoma

## Abstract

Telmisartan, an angiotensin II type 1 receptor (AT1R) antagonist, possesses cytotoxic activity towards BRAF-mutated melanoma cell lines. However, its antihypertensive effects limit its use in the population of normotensive patients. To mitigate this shortcoming, a group of eight telmisartan–amino acid conjugates, designed to have reduced or no AT1R affinity with enhanced cellular uptake, were synthesized by the coupling reaction in yields ranging from 34% to 60%. Their cytotoxicity was tested on BRAF V600E-mutated melanoma cell lines (A375 and 518A2), and compounds **1**, **3**, and **8** stood out as the best candidates. These three compounds were also tested on the vemurafenib-resistant (A375R) and normal (HaCaT and MRC-5) cell lines, and compound **8** showed better cytotoxicity (IC_50_ = 8.84 ± 1.24 µM) and selectivity (>3.50) when compared to telmisartan (IC_50_ = 29.23 ± 3.88, selectivity > 2.40). The cellular uptake of compounds **1** and **8** was significantly higher than telmisartan, with substantial accumulation in the membrane and nuclear compartments. Unlike telmisartan, compounds **1**, **3**, and **8** did not inhibit angiotensin II-induced Ca^2+^ signaling, which indicates diminished AT1R binding. All three compounds induced cell cycle arrest and disrupted mitochondrial morphology and membrane potential. These findings highlight their potential as non-antihypertensive telmisartan derivatives for melanoma therapy.

## 1. Introduction

Compared to other types of cancer, skin melanoma is fairly common and is estimated to account for approximately 5.1% of new cases of all types of cancer in 2025 [1]. Approximately fifty percent of cutaneous melanomas harbor the BRAF V600E mutation, which is characterized by constitutive kinase activity, aggressive behavior, and poor prognosis. Targeted therapy with BRAF and MEK inhibitors has become the standard treatment of patients with BRAF V600 mutation-positive melanoma with great efficacy, especially when used in combination [2,3,4]. Despite the high response rates to combination therapy, the development of resistance continues to increase. The mechanisms of resistance to inhibition of the BRAF signaling pathway have been elucidated by the metabolic plasticity of melanoma and reprogramming from glycolysis to mitochondrial metabolism [5,6,7,8].

Metabolic reprogramming in carcinoma cells necessitates the inclusion of alternative therapeutic approaches. Therefore, there is a clear need for further research into novel drugs that target various aspects of cellular metabolism in cancer cells [8,9]. Due to all the difficulties tied to developing new anti-cancer drugs (e.g., time-consuming process and escalating costs with a high risk of failure), drug repositioning is becoming an increasingly significant and more accessible alternative strategy in the ongoing search for innovative cancer treatments. Drug repositioning, also known as drug repurposing or “old drugs for new uses”, is a research and development strategy that involves identifying new therapeutic applications for already approved or previously investigated drugs [10,11,12]. Recent studies have identified a wide range of drugs from various pharmacological classes that have effects different from their original purpose and can be repurposed in the treatment of specific types of cancer. Successful drug repurposing has been demonstrated with the use of several antipsychotic, antimalarial, antidiabetic, and antiviral drugs (sertraline, gemcitabine, metformin, clotrimazole, etc.) in the field of oncology [13,14].

AT1R antagonists (sartans), in addition to their use in the treatment of hypertension, chronic heart failure, and chronic kidney disease, could also affect the progression and outcome of the disease in people with malignancy [15]. There are many in vitro and in vivo studies that indicate the use of AT1R antagonists could be associated with a decrease in the incidence of various types of cancer. Recent experimental data suggest that angiotensin-converting enzyme (ACE) inhibitors and AT1R antagonists have a suppressive effect on tumor progression through the normalization of the tumor vasculature, decreases in the deposition of the extracellular matrix, and the modulation of the tumor immune microenvironment [16,17,18]. It has been pointed out that the use of AT1R antagonists is associated with a lower incidence rate of tumor occurrence and an improved effect in reducing mortality in patients with different types of cancer [19,20,21,22,23].

It was shown that the antihypertensive drug telmisartan (AT1R antagonist and partial PPARγ agonist), but not losartan (pure AT1R antagonist), has a cytotoxic effect on BRAF V600E melanoma cells, as well as on cells resistant to a BRAF inhibitor, with the effect on mitochondrial integrity and on the induction of reactive oxygen species production [5]. Hence, telmisartan could potentially be repurposed for melanoma therapy [5,10,24]. However, the doses necessary to achieve anti-melanoma effects in patients were in the reported range for the antihypertensive effects, presenting an obstacle in normotensive and hypotensive cancer patients, and limiting the repurposing potential. According to the International Agency for Research on Cancer (IARC), arterial hypertension is not universally observed among all cancer patients, highlighting the importance of developing new drugs with anticancer activity but without antihypertensive effect [25]. Therefore, telmisartan modifications that would limit the AT1R activity while maintaining the cytotoxic effects are of great interest.

Cancer cells utilize amino acids (AAs) to obtain energy and as a source to modulate growth signaling [26,27]. As cancer cells are unable to synthesize essential AAs, they take them up from the extracellular environment, utilizing AA transporters on the surface, to meet increased metabolic needs [28,29]. Accordingly, a growing number of preclinical studies have demonstrated the utility of conjugating AAs to therapeutic agents, enabling transporter-mediated targeted drug delivery and thereby enhancing the uptake of lead drugs into cancer tissue [30,31,32]. Interestingly, melanoma cells have specific amino acid dependencies, with tyrosine and phenylalanine modulating melanoma cell behavior [33]. Therefore, the incorporation of amino acids into the side chain of the telmisartan structure could have several advantages. First, the addition of AAs is expected to significantly increase the cellular uptake of new telmisartan derivatives into the cancer cells and enhance the anticancer activity of telmisartan [25,34,35]. Second, bulkier AAs may decrease AT1R binding, thus circumventing the antihypertensive effects, and lastly, the addition of specific AAs might additionally modulate melanoma cell behavior.

The aim of this study was to synthesize new telmisartan–amino acid conjugates that lack antihypertensive activity, and to investigate their anticancer potential against BRAF V600-mutated melanoma cells. The choice of amino acids was guided by the goal of designing compounds with a reduced likelihood of binding to the AT1R, thereby minimizing antihypertensive side effects.

## 2. Results

### 2.1. Molecular Docking

Fifteen telmisartan–amino acid conjugates were designed with glycine, L-alanine, L-phenylalanine, L-histidine, beta-alanine, gamma-aminobutyric acid, L-tryptophan, N-methyl-L-tryptophan, L-isoleucine, L-leucine, L-tyrosine, L-valine, L-glutamic acid, L-proline, L-taurine, and L-valine. Their docking scores are presented in Table 1.

**Figure 1:**
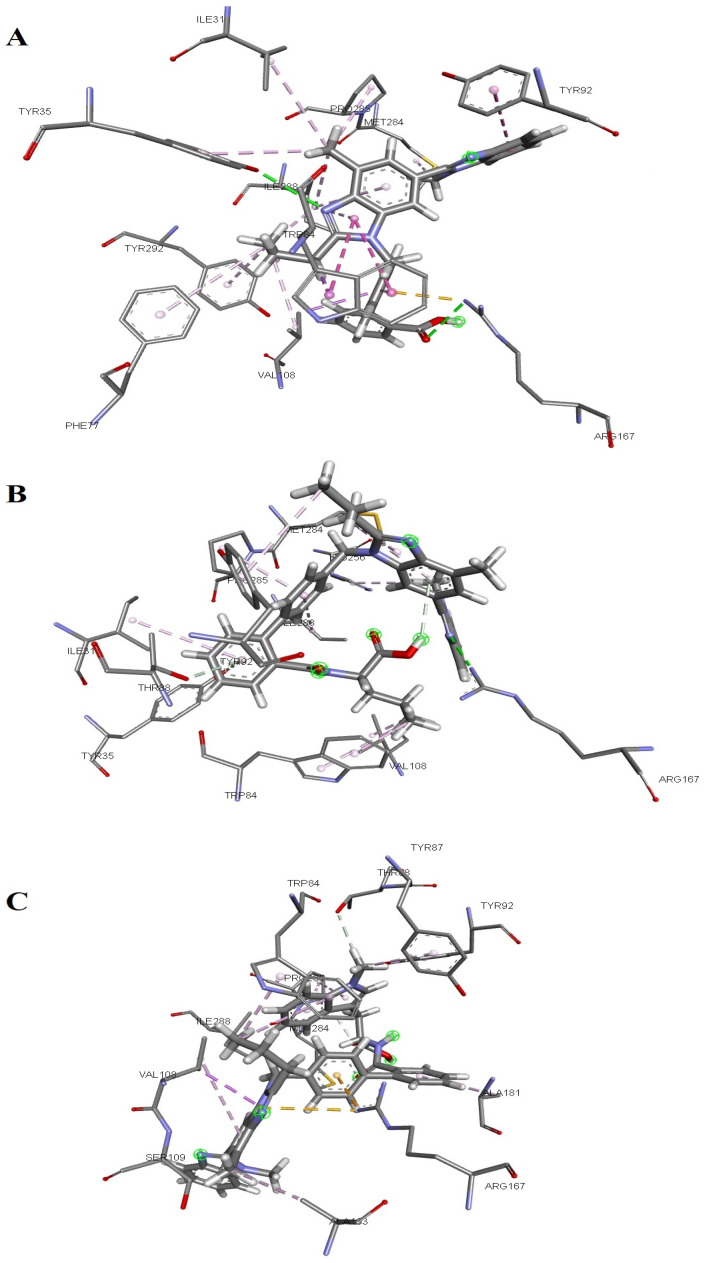
Molecular docking analysis of binding to AT1 receptor (**A**) telmisartan, (**B**) telmisartan–L-leucine conjugate, (**C**) telmisartan–N-methyl-L-tryptophan conjugate. Green lines: hydrogen bonds, orange lines: π–cation interactions, dark pink lines: π–π interactions, pale pink lines: π–alkyl interactions, orange lines: π–cation interactions.

Telmisartan formed two hydrogen bonds with the AT1R (carboxylic group of telmisartan interacts with ARG167, while its nitrogen atom from the central benzimidazole ring interacts with TYR35). Apart from these two interactions, both benzimidazole rings form several pi–sigma or pi–alkyl interactions with TYR92, MET284, ILE31, PRO285, ILE288, TRP84, VAL108, PHE77, and TYR292 (Figure 1A). The docking of telmisartan revealed interactions with the AT1 receptor, which had been previously identified as important for the AT1 antagonistic activity—two hydrogen bonds (Arg167 with –COOH, Tyr35 with =N), and hydrophobic interaction with Trp84 [36].

Among designed compounds, telmisartan–glycine and telmisartan–L-leucine (Figure 1B) conjugates do not form interactions with TYR35 and TRP84 (in addition, telmisartan–L-leucine is oriented in the receptor differently compared to telmisartan, so its terminal benzimidazole ring forms a hydrogen bond with ARG167 and not the carboxylic group). Telmisartan–gamma-aminobutyric acid conjugate does not form an interaction with TYR35, while telmisartan–L-isoleucine conjugate does not form an interaction with TRP84. These compounds also showed significantly higher docking scores in comparison to telmisartan (Table 1). The binding of several designed compounds into the AT1 receptor (telmisartan–L-phenylalanine, telmisartan–N-methyl-L-tryptophan, telmisartan–L-tryptophan, and telmisartan–L-histidine) is characterized by unfavorable interactions, which occur because of their voluminosity (Figure 1C). These compounds showed the highest docking scores among all designed conjugates.

All designed derivatives could be classified into three groups according to calculated docking scores and chemical structures. Only conjugates of telmisartan with glycine and beta-alanine showed similar docking scores to telmisartan (less than 2 units difference), but some important interactions with the receptor were not observed (the first group). Other conjugates had much higher docking scores, indicating less favorable binding in comparison to telmisartan, and could be divided into two groups: conjugates with aliphatic amino acids (the second group) and conjugates with aromatic and heterocyclic amino acids (the third group). The highest docking scores were calculated for telmisartan conjugates with L-phenylalanine, L-tryptophan, N-methyl-L-tryptophan, L-tyrosine, and L-histidine (the third group), which also showed the highest number of unfavorable interactions.

Representative compounds from each of the three groups were selected for synthesis: N-methyl-L-tryptophan (compound **1**), L-histidine (compound **2**), L-tyrosine (compound **3**), L-isoleucine (compound **4**), glycine (compound **5**), gamma aminobutyric acid (compound **6**), L-leucine (compound **7**), and L-phenylalanine (compound **8**).

### 2.2. Synthesis

Designed compounds (**1**–**8**) represent amides of telmisartan and selected AAs. In the first step, telmisartan amides were synthesized with the corresponding amino acid methyl esters via a coupling reaction using DCC as the coupling agent (compounds **1a**–**8a**). In the second step, the methyl ester groups in compounds **1a**–**8a** were hydrolyzed using an aqueous solution of sodium hydroxide. The resulting mixtures were then purified by preparative TLC to afford the final compounds (**1**–**8**) in yields ranging from 34.0% to 60.0%.

### 2.3. Newly Synthesized Telmisartan Derivatives Impair Melanoma Cell Viability

The effects of compounds **1**–**8** and telmisartan on the viability of two different malignant melanoma cell lines, A375 and 518A2, were analyzed using the MTT assay. After 72 h of treatment, the IC_50_ values were determined, as shown in Table 2. These results identified three particularly effective compounds that exhibited approximately twofold higher potency compared to telmisartan.

For the three most potent compounds (**1**, **3**, and **8**), the effect on the viability of vemurafenib-resistant A375R melanoma cells and two non-malignant cell lines, HaCaT (human keratinocytes) and MRC-5 (fetal fibroblasts), was examined (Table 3). The obtained results indicated that compounds **1**, **3**, and **8** were effective in the vemurafenib-resistant melanoma cell line as well (Table 3). Based on the IC_50_ values against the normal cell lines, compound **8** demonstrated the best selectivity.

**Figure 2:**
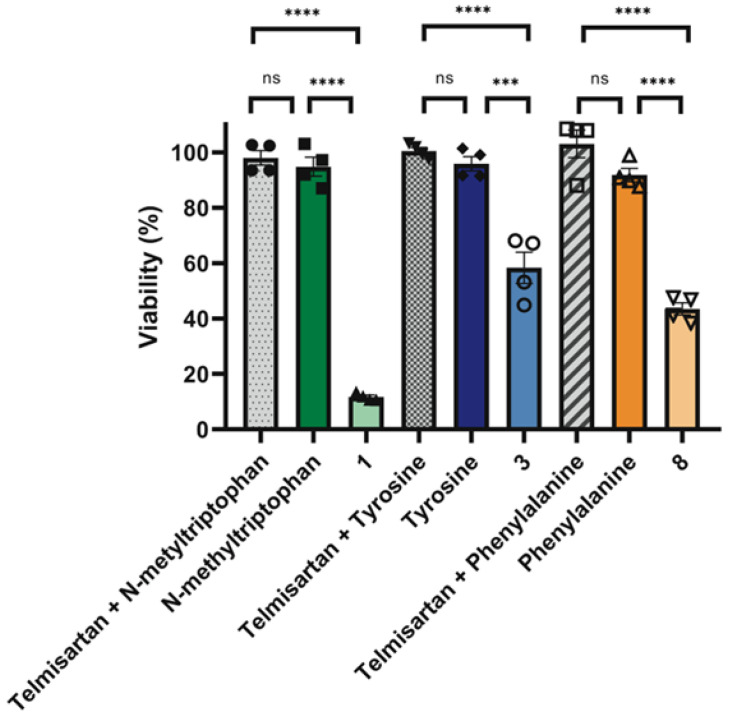
Effect of compounds **1**, **3**, and **8**, amino acids alone, and combinations of telmisartan with amino acids on A375 cell viability. Shown are mean ± SEM. Analyzed with one-way ANOVA with post-hoc Tukey’s test from four independent experiments *** *p* < 0.001, **** *p* < 0.0001, ns—non-significant.

Next, we sought to determine whether the observed effects were attributable to the amino acid components of the compounds. A375 cells were treated with the combinations of telmisartan and corresponding amino acid (N-methyl-L-tryptophan, L-tyrosine, or L-phenylalanine), amino acids alone, and tested compounds (**1**, **3**, and **8**). Compounds were prepared in a concentration of 25 μM (compound **1** corresponding to 18 μM telmisartan and 7.5 μM N-methyl-L-tryptophan, compound **3** corresponding to 19.5 μM telmisartan and 6.25 μM L-tyrosine, and compound **8** corresponding to 19 μM telmisartan and 6.5 μM L-phenylalanine) and compared to 18 to 19.5 μM telmisartan in combination with 6.25 to 7.5 μM AAs (Section 4). Neither the amino acids alone nor their combinations with telmisartan affected cell viability more than 10% at the applied dose after 72 h of treatment, while significant reductions in cell viability were observed only after treatment with compounds **1**, **3**, and **8** (Figure 2).

### 2.4. Cellular Uptake and Distribution of Tested Compounds

Given the increased efficacy of compounds **1**, **3**, and **8** in viability assays, we hypothesized that these compounds can accumulate in melanoma cells to a greater extent than telmisartan itself. To test this hypothesis, A375 cells were treated with 25 μM telmisartan or compounds **1**, **3**, and **8** for 24 h, and then the compound content was measured in the whole cell lysates and cell fractions by mass spectrometry. Compound content in whole cell lysates, cytoplasmic, membrane, soluble, and chromatin-bound nuclear extracts was expressed as μmol of compound per μg of cell protein and normalized to telmisartan (Table 4).

All three tested compounds accumulated in A375 cells to a greater extent than telmisartan (up to 12 times more for compound **1**). The highest accumulation of telmisartan and compounds **1**, **3**, and **8** in subcellular compartments was in the membrane fraction (ranging from 0.197 μmol/L for telmisartan to 26.814 μmol/L for compound **8**). While compound **3** accumulated to a lesser extent than telmisartan in the cytoskeletal and nuclear fractions, compounds **1** and **8** accumulated more than telmisartan in all the examined fractions, with a several hundred-fold increase in the membrane fraction.

### 2.5. Angiotensin II-Induced Ca^2+^ Release Is Intact in the Presence of Compounds ***1***, ***3***, and ***8***

**Figure 3:**
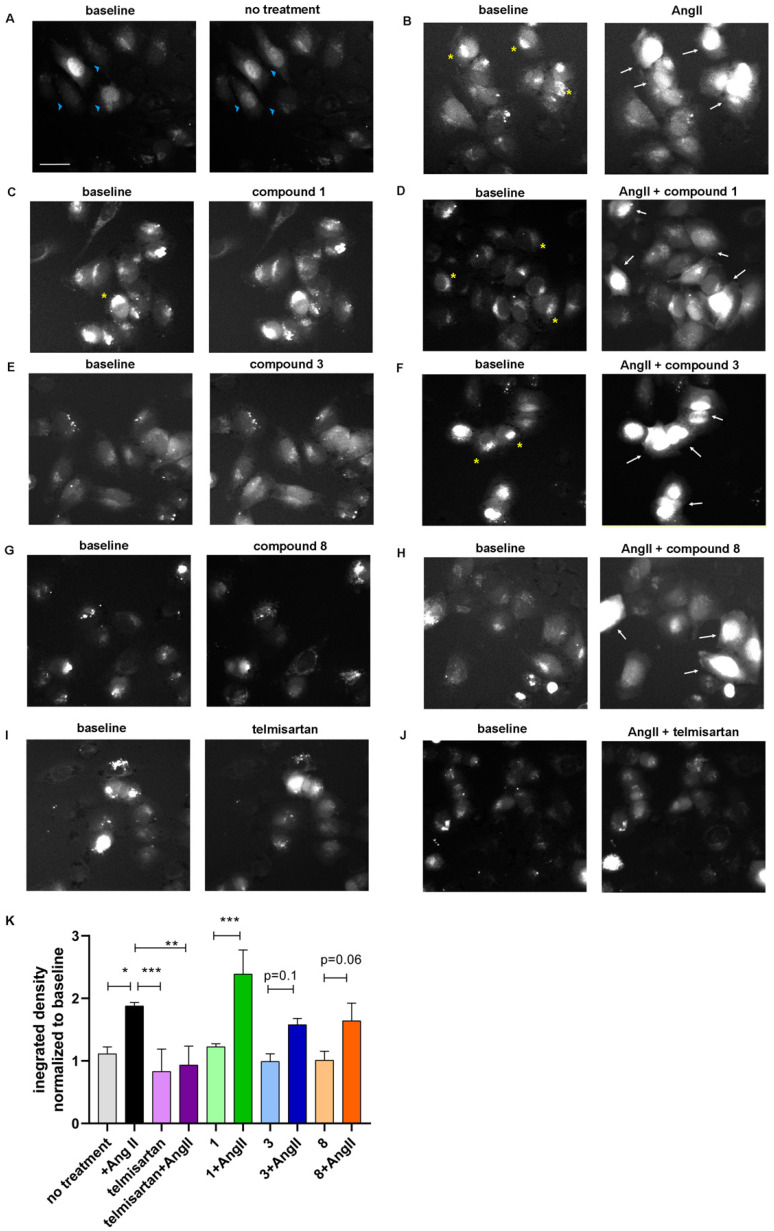
Ca^2+^ distribution in A375 cells upon treatment with AngII and compounds **1**, **3**, and **8** (**A**–**J**). Blue arrowheads: baseline fluorescence in the cytoplasm and nuclei. Yellow asterisk: intracellular calcium stores. White arrows: AngII-induced fluorescence burst. Scale bar 20 μm. (**K**) Quantification of the fluorescence integrated density from three independent experiments. Shown are mean and SD. Analyzed with One-way ANOVA and Tukey’s multiple comparison test. * *p* < 0.05. ** *p* < 0.01. *** *p* < 0.001.

To test whether the cellular response to AngII is intact in the presence of compounds **1**, **3**, and **8**, and indirectly that they do not bind to the AT1R, a cellular signaling cascade downstream of AT1R activation was examined. Angiotensin II binding to AT1R induces a dose-dependent Ca^2+^ release from the internal Ca^2+^ stores [37], which can be abolished with AT1R blockers [38]. Upon AngII treatment, about 60% of bound Ca^2+^ is rapidly released in the first 2 min of stimulation [39]. To examine whether telmisartan derivatives can prevent AngII-induced Ca^2+^ release, a pharmacological concentration of 1 μM AngII was used [40] to elicit cytosolic Ca^2+^ and nuclear Ca^2+^ surge [41] and measured it in the presence of 25 μM AT1R blocker telmisartan or the compounds **1**, **3**, and **8**. A375 cells were stained with the Fluo-4 AM calcium indicator, which increases fluorescence intensity upon Ca^2+^ binding, and imaged after 2 min of treatment (Figure 3, left panel baseline 0 min, right panel treatment 2 min). Ca^2+^ is the most versatile cellular messenger [42], and melanoma cells use oscillatory waves of Ca^2+^ signaling to orchestrate responses to extrinsic and intrinsic stimuli [43,44,45]. Baseline Ca^2+^ signal in untreated A375 cells can be seen in Figure 3A, with nuclei turning “on” and “off”. Upon AngII treatment, Ca^2+^ is released from the internal stores (labeled with a yellow asterisk) and can be seen as increased fluorescence in the cytoplasm and nuclei (Figure 3B, white arrows, Figure 3K quantification). Upon treatment with the compounds **1**, **3**, or **8** alone, there were no significant changes in the baseline Ca^2+^ signaling (Figure 3C,E,G), while in the combination treatment of compounds **1**, **3**, or **8** and AngII, compounds in 25 μM concentration did not prevent AngII-induced Ca^2+^ increase in the cytoplasm and nuclei (Figure 3D,F,H, white arrows, Figure 3K quantification). On the contrary, 25 μM telmisartan prevented AngII-induced Ca^2+^ increase in the combination treatment (Figure 3J). These results imply that compounds **1**, **3**, and **8** do not prevent AngII-induced Ca^2+^ signaling and, indirectly, that—unlike telmisartan—they do not bind to or block AT1R.

### 2.6. Effects of the Novel Telmisartan Derivatives on the Melanoma Cell Cycle

**Figure 4:**
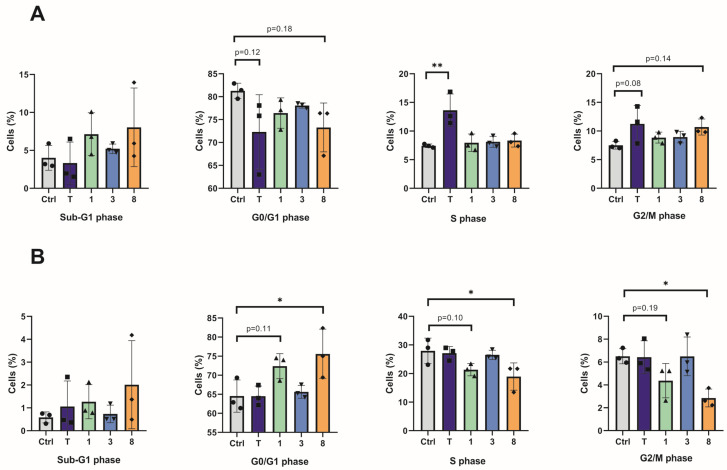
Effect of telmisartan and compounds **1**, **3**, and **8** on the cell cycle distribution in A375 (**A**) and A375R (**B**) cells. Shown are mean and SD. Analyzed with One-way ANOVA and Dunnett’s multiple comparison test from three independent experiments (Mean ± SD); * *p* < 0.05, ** *p* < 0.01.

As it was previously reported that telmisartan can induce cell cycle arrest in G0/G1 [46,47] or G2/M phase [48] depending on the cancer cell line, we next determined whether the novel derivatives retain this ability, even though they do not have the AT1R inhibiting function. After 72 h of treatment with 25 µM of compounds **1**, **3**, and **8**, distinct alterations in cell cycle distribution were observed in both A375 and A375R cell lines (Figure 4). In A375 cells, treatment with compounds **1**, **3**, and **8** resulted in the increased accumulation of cells in the subG1 phase, albeit not statistically significant (Figure 4A). In the vemurafenib-resistant A375R cells, which have higher proliferation capacity than A375 cells, treatment with compounds **1** and **8** caused cell accumulation in the G0/G1 phase, suggesting an inhibition of the cell cycle progression at the initial checkpoint (Figure 4B), with a corresponding decrease in S and G2/M cell distribution. Meanwhile, cells treated with telmisartan or compound **3** exhibited a cell cycle profile comparable to that of the untreated control cells.

### 2.7. Compounds ***1***, ***3***, and ***8*** Impair Mitochondrial Function in Melanoma Cells

**Figure 5:**
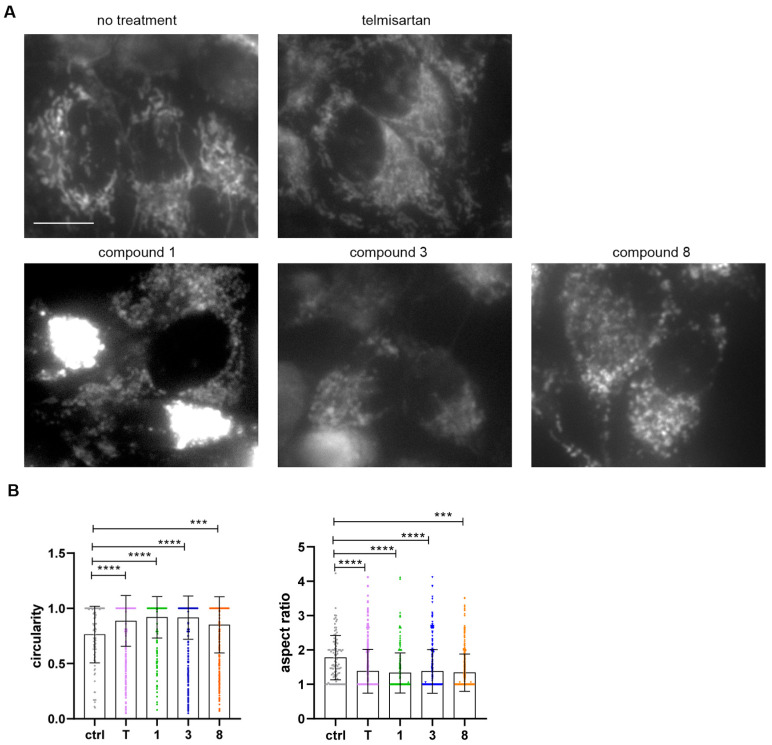
(**A**) A375 cells mitochondrial morphology after 24 h treatment with telmisartan or compounds **1**, **3**, and **8**. Scale bar 10 μm. (**B**) Quantification of the mitochondrial circularity and aspect ratio with Image J64 V1.47 Analyze particles tool. Shown are mean and SD. Analyzed with One-way ANOVA and Dunnett’s multiple comparison test. *** *p* < 0.001, **** *p* < 0.0001.

We have previously reported that telmisartan induces mitochondrial fission in melanoma cells, thus inducing cell death [5]. Therefore, we next examined whether decreased viability in cells treated with compounds **1**, **3**, and **8** was due to mitochondrial disruption. A375 cells were treated with 25 μM telmisartan or compounds **1**, **3**, and **8** for 24 h, and live mitochondria were stained with MitoTracker Green FM and observed under the microscope. All three compounds disrupted the mitochondrial network to a greater extent than telmisartan, with compounds **1** and **8** inducing dramatic fission and clumping of mitochondria (Figure 5A). Mitochondria had higher circularity and a decreased length-to-width ratio under all treatments compared to the control (Figure 5B).

**Figure 6:**
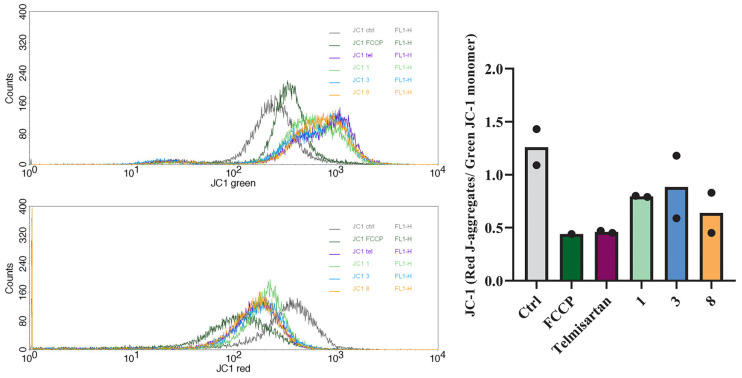
JC-1 fluorescence of red aggregates and green monomers ((**left**) panels) and their ratio ((**right**) panel) in A375 cells after 24 h treatment with telmisartan and compounds **1**, **3**, and **8**. Shown are individual values from two independent experiments.

As such a dramatic structural change implies impaired mitochondrial function, mitochondrial potential (ΔΨm) was subsequently measured in treated cells with the JC-1 assay. JC-1 dye accumulates in mitochondria in a potential-dependent manner, with red-fluorescent aggregates in polarized mitochondria and green-fluorescent monomers in depolarized mitochondria. The ratio of red JC-1 aggregates to green JC-1 monomers decreased in all treated groups compared to the untreated control, indicating mitochondrial depolarization (Figure 6). FCCP was used as a positive control for depolarization and showed the lowest red/green ratio. These findings suggest that compounds **1**, **3**, and **8** retain the telmisartan ability to impair mitochondrial function in melanoma cells.

### 2.8. Stability in Biological Media

To test stability in biological media, the best drug candidates (compounds **1**, **3**, and **8**) were incubated in 0.05 M hydrochloric acid during 2 h (this medium simulates the acidic stomach environment and 2 h can be considered the average retention of drugs in the stomach after an oral administration), in phosphate buffer pH 5.5 during 5 h (this medium simulates the average intestinal pH value, which is the most significant absorption site of the majority of drugs) and in RPMI 1640 medium during 72 h (this medium is used for biological experiments and 72 h corresponds to the longest exposure of tested compounds in this study). The greatest change in concentration after incubation in 0.05 M hydrochloric acid was observed for compound **8** (3.04%), in phosphate buffer pH 5.5 for compound **3** (3.49%), and in RPMI 1640 medium for compound **8** (2.00%). The obtained results indicate good stability of these three compounds in the tested media.

## 3. Discussion

Anticancer properties of the AT1 receptor antagonist telmisartan have been extensively studied over the past decade with the intent of repurposing in oncology. Through various mechanisms, including cell cycle arrest and caspase-dependent apoptosis, anticancer effects of telmisartan were shown in cholangiocarcinoma [49], endometrial cancer [50], glioma cells [46], esophageal squamous cell carcinoma [51], gastric cancer cells [52], gastrointestinal stromal tumor [53], non-small cell lung cancer [54,55], melanoma cells [5], ovarian cancer cells [56], and adult T-cell leukemia cells [57].

Other AT1 receptor antagonists do not induce cancer cell death [5,57], implying that AT1 receptor binding ability is not prerequisite for anticancer effects. This is of importance, as one of the possible drawbacks in repurposing telmisartan in oncology is the administration of high micromolar dosages needed to achieve anticancer effects. After the oral intake of 320 mg of telmisartan in males, the maximum steady-state plasma level is around 20 μM [58], and the plasma concentrations of telmisartan are generally two to three times higher in females [59]. Therefore, effective anti-cancer doses of around 50 μM of telmisartan could be administered to patients. However, these may pose significant clinical consequences of lowering blood pressure due to its high potency for the AT1 receptor.

Second, like other small-molecule drugs, telmisartan also shows limited solubility and dose-limiting toxicities. Several lines of work have been applied to overcome these obstacles. First is the synthesis of telmisartan derivatives that have lower affinities for the AT1 receptor. The synthesis of alkylamine telmisartan derivatives with reduced AT1 antagonistic activity but increased anticancer activity has been reported, with higher growth inhibition in the MDA-MB-231 tumor xenograft mouse model compared to telmisartan [25]. The heterocyclic core of telmisartan was shown to be important for the cell death-modulating effects of telmisartan-derived compounds and most recently, the carboxamide derivative of telmisartan was reported to reduce the number of therapy-resistant cancer stem cells and tumor growth in an imatinib-resistant leukemia xenograft model [35]. Further efforts to increase stability of telmisartan derivatives in biological environments through structural modifications are also underway [60], as well as development of nano-formulations of telmisartan by encapsulating it inside cell-derived extracellular vesicles and bio-mimetic lipid nanovesicles [61], to increase its delivery.

We have previously reported that telmisartan induces mitochondrial disruption in melanoma cells, making it a promising candidate for targeting vemurafenib-resistant melanoma cells that heavily rely on mitochondrial metabolism [5]. Therefore, the aim of the current study was to develop telmisartan derivatives that would have decreased AT1 receptor binding affinity (and consequently reduced AT1 antagonistic activity), thus limiting the antihypertensive effects while maintaining the cytotoxic effects exerted through cancer cell mitochondrial targeting.

The introduction of amino acids should enhance water solubility and facilitate transport across cell membranes through specialized transport systems. One such example is the L-type amino acid transporter, which mediates the uptake of large neutral amino acids such as L-phenylalanine [62]. Importantly, our experiments confirmed that the amino acids themselves were non-toxic at the concentrations used. Therefore, amino acids were selected for the design of new telmisartan derivatives. Zhang et al. co-crystalized the AT1 receptor with its selective antagonist ZD7155. The residues shown by mutagenesis to be critical for the binding for AT1R were ARG167, TYR35 and TRP84 [36]. Our docking study performed on telmisartan also identified interactions with these three amino acids. Therefore, the loss of AT1 antagonistic activity might be achieved by chemical modifications of telmisartan that will lead to a molecular geometry change, which will further result in the elimination of interactions with these three amino acids. Since the mechanism of telmisartan anticancer activity is not completely resolved, the main idea underlying the design of new compounds was to make small changes that, hopefully, will not negatively affect anticancer activity, but will cause molecular geometry to change enough to result in the loss or decrease in AT1 receptor affinity and thus antihypertensive activity. Among the designed compounds from which a lack of or lower AT1 antagonistic activity could be expected, two groups were identified: compounds that lack at least one important binding interaction (e.g., telmisartan conjugate with glycine) and compounds that cannot access the AT1 receptor site due to steric factors (e.g., telmisartan conjugates with L-phenylalanine, L-tyrosine, and N-methyl-L-tryptophan).

The synthesis of selected designed conjugates was performed in two steps. The first step was the conjugation of telmisartan with the methyl ester of the corresponding amino acid using DCC. This step was necessary because direct conjugation of the carboxylic group of telmisartan with the amino group of amino acids is difficult due to their poor reactivity. Amino acids exist as zwitterions, where the amino group is protonated, reducing its nucleophilicity [63,64]. This type of reaction can also be carried out using 1-ethyl-3-(3-dimethylaminopropyl)carbodiimide (EDC), which is generally considered more convenient, as its urea by-product is water-soluble and can be removed by simple aqueous washes, thereby avoiding additional purification steps such as chromatography. The second step was the hydrolysis of methyl esters to obtain the free carboxylic group. Although it was observed that hydrolysis of an ester group in a water/THF two-phase system is more efficient in the presence of lithium ions [65], our preliminary results indicated similar efficiency when using either LiOH or NaOH.

Through a battery of in vitro tests, we have found that compounds **1**, **3**, and **8** (telmisartan conjugates with N-methyl-L-tryptophan, L-tyrosine, and L-phenylalanine, respectively) have lower IC_50_ values than telmisartan in melanoma cell viability assays and that they disrupt the mitochondrial network to a greater extent than telmisartan at the same dose. These effects were, in part, due to the greater intracellular accumulation of compounds, but their other anticancer properties remain to be explored. Most promising for further testing was compound **8** (telmisartan conjugate with L-phenylalanine), which showed the best selectivity towards cancer cells, accumulated more than 270-fold compared to telmisartan in the cellular membrane fraction (where mitochondria are located), and induced dramatic mitochondrial fission, while leaving AT1R calcium signaling intact. This compound is therefore the best candidate for further general tolerability and tumor growth in vivo studies.

Taken together, aromatic amino acid conjugation with telmisartan proved a promising strategy for increased cell accumulation and increased cytotoxic effects of telmisartan. Given that L-phenylalanine, L-tyrosine, and N-methyl-L-tryptophan are important in protein synthesis and the generation of a variety of secondary metabolites, cancer cells are often in high demand for these amino acids. Our future efforts will entail delineating the possible effects on cancer cell metabolism by the three most potent telmisartan derivatives.

## 4. Materials and Methods

### 4.1. Molecular Docking

OMEGA 2.5.1.4 software [66,67] was used for the pre-generation of ligand conformations, while molecular docking analysis was performed in OpenEye’s OEDocking 3.2.0.2 software [68,69,70], using the fast rigid exhaustive docking (FRED) tool. The docking poses were scored using the Chemgauss4 scoring function [71].

Molecular docking analyses were performed to examine the binding interactions of telmisartan and designed Telmisartan–Amino acid conjugates with AT1 receptor. For this purpose, the crystal structure of AT1 receptor in complex with selective inhibitor ZD7155 (pdb code: 4yay) was retrieved from the Protein Data Bank (PDB) [72]. The receptor structure was prepared for docking in MAKE Receptor 3.2.0.2 software [73]. Molecular docking analyses were conducted within grid boxes centered around the co-crystallized ligands, with dimensions of 17.33 Å × 22.33 Å × 18.67 Å. The box volume was 7226 Å^3^. Docking site shape parameters were set to a balanced configuration.

To validate the docking protocol, the native conformations of ligand were re-docked into the active sites of AT1 receptor. The docking-generated conformers of the co-crystallized ligand were then aligned with corresponding experimental crystal structure, and root mean square deviation (RMSD) value of the most similar conformation was below 2.0 Å, confirming the reliability of the docking approach used in this study.

### 4.2. Synthesis

#### 4.2.1. Chemicals

Telmisartan was purchased from Fisher Scientific (Loughborough, UK). Methyl esters of glycine, L-leucine, L-isoleucine, L-phenylalanine, N-methyl-L-tryptophan, gamma-aminobutyric acid, L-histidine, and L-tyrosine, as well as sodium dihydrogen phosphate dihydrate, disodium hydrogen phosphate, and ortho-phosphoric acid were purchased from Sigma Aldrich (Steinheim, Germany).

N-hydroxybenzotriazole (HOBt) and *N*,*N*-dimethylformamide (DMF) were purchased from Sigma Aldrich (Steinheim, Germany), whereas triethylamine (TEA) was purchased from Acros Organics (Geel, Belgium). Tetrahydrofuran (THF), *N*,*N*-dicyclohexylcarbodiimide (DCC), and dimethyl sulfoxide (DMSO) were purchased from Fluka Chemie GmbH (Bruch, Switzerland). Chloroform and methanol were purchased from JT Baker (Loughborough, UK). Sodium hydroxide 0.1 M (analytical volumetric solution) was purchased from LabExpert (Ljubljana, Slovenia). Silica gel for preparative thin-layer chromatography was purchased from Merck (Darmstadt, Germany).

#### 4.2.2. General Procedure for the Synthesis of Novel Telmisartan Derivatives

**Figure 7:**
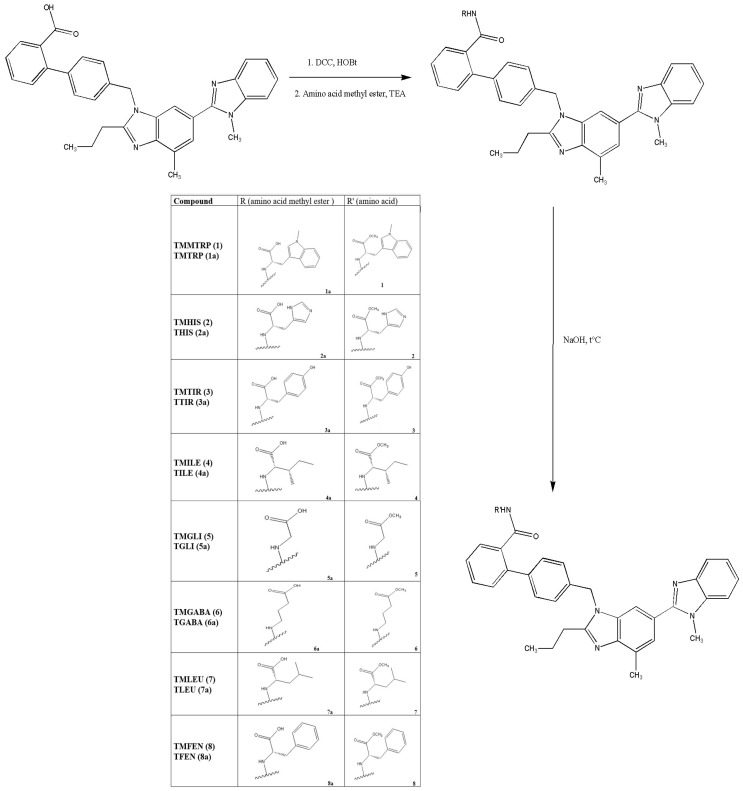
Synthesis of telmisartan–amino acid conjugates.

Synthesis of presented compounds was performed in two steps (Figure 7). In the first step, telmisartan (0.10 mmol, 1 eq) was dissolved in a mixture of DMF, chloroform, and DMSO with constant stirring in an ice bath. Subsequently, DCC (0.20 mmol, 2 eq) and HOBt (0.15 mmol, 1.5 eq) were added. The mixture was stirred and cooled in an ice bath for 1 h. Then, the reaction mixture was left overnight without stirring at a temperature below 8 °C. Corresponding ester of amino acid (as a hydrochloride salt, 0.1 mmol, 1 eq) was dissolved in DMF, TEA was added (2 eq), and this mixture was cooled in an ice bath for 1 h (amino acid solution). Then, the mixture of telmisartan, DCC, and HOBt was filtered through a membrane filter (0.45 µm) and added dropwise to the amino acid solution. This reaction mixture was stirred at room temperature for next 5 h, and after that, evaporated to dryness. Ultimately, obtained residue was purified by preparative thin-layer chromatography. Synthesized intermediates (telmisartan—methyl ester of amino acids conjugates, **1a**–**8a**) were purified using preparative TLC. The mobile phases used for purification were chloroform/methanol 90:10 *v*/*v* (**3a**, **5a**, **6a**, and **8a**), chloroform/methanol 93:7 *v*/*v* (**1a**, **4a**, and **7a**), and chloroform/methanol 85:15 *v*/*v* (**2a**). Recrystallization of purified intermediates (**1a**–**8a**) was performed in the mixture of chloroform/methanol 70/30 (*v*/*v*).

In the second step, **1a**–**8a** were dissolved in THF. Then, 0.1 M NaOH was added dropwise so that the molar ratio was 1:25 (compounds **1a**–**8a**: NaOH). The reaction mixture was stirred and heated for 1 h, and after that, it was evaporated to dryness and purified by preparative TLC to obtain telmisartan—amino acid conjugates (compounds **1**–**8**). The mobile phases used for purification were chloroform/methanol 85:15 *v*/*v* (**1**, **4**, **6**, **7**, **8**), chloroform/methanol 80:20 *v*/*v* (**3**), chloroform/methanol 70:30 *v*/*v* (**5**), and chloroform/methanol 40:60 *v*/*v* (**2**). Recrystallization of purified compounds was performed in the mixture chloroform/methanol 50/50 (*v*/*v*).

#### 4.2.3. Structural Characterization

Synthesized telmisartan—amino acid conjugates (compounds **1**–**8**) were structurally characterized by determining melting points and by spectroscopic methods (IR, NMR, MS/MS, and HRMS). Melting points were determined on Boetius PHMK 05 apparatus (Bremen, Germany). IR spectra were recorded using FT-IR spectrometer, Nicolet iS10 (Thermo Fisher Scientific, Madison, WI, USA). NMR spectra were recorded on NMR BRUKER AVANCE III 400 (Bruker Biospin GmbH, Rheinstetten, Germany). MS/MS analyses were performed using TSQ Quantum Access MAX triple quadrupole mass spectrometer (Thermo Fisher Scientific, San Jose, CA, USA), equipped with heated electrospray ionization source. Determination of exact masses was performed using LTQ Orbitrap XL Mass Spectrometer (Thermo Fisher Scientific, Bremen, Germany).

2-{[4′-(1,7′-dimethyl-2′-propyl-1H-[2,5′]bibenzoimidazolyl-3′-ylmethyl)-biphenyl-2-carbonyl]-amino}-3-(1-methyl-1H-indol-3-yl)-propionic acid (compound **1**):

White crystalline solid. Yield 53.3%. Melting point: 190.8–195 °C. IR (ATR) ν_max_ (cm^−1^): 740.63, 850.23, 1154.24, 1282.29, 1323.58, 1438.50, 1594.06, 2918.02, 3383.17. ^1^H NMR (400 MHz, DMSO) δ ppm: 8.02 (1H, s, -COOH), 6.82–7.53 (19H, m, ArH), 5.46 (2H, s, -CH_2_- at position C3′), 4.35 (1H, s, -CH from the amino acid side chain), 3.76 (3H, s, -CH_3_ at position N1), 3.53 (3H, s, -CH_3_ from the amino acid side chain), 3.19–3.22 and 2.98–3.01 (2H, m, -CH_2_ from the amino acid side chain), 2.83 (2H, m, -CH_2_-CH_2_-CH_3_), 2.65 (3H, s, -CH_3_ at position C7′), 1.77 (2H, m, -CH_2_-CH_2_-CH_3_), 0.95 (3H, t, *J* = 7.2 Hz, -CH_2_-CH_2_-CH_3_). ^13^C NMR (100 MHz, DMSO) δ ppm: 168.80, 156.65, 154.47, 143.10, 142.89, 139.69, 138.92, 137.31, 137.08, 136.86, 136.00, 135.20, 130.37, 129.89, 129.04, 128.74, 128.36, 128.20, 127.41, 126.62, 123.74, 123.70, 122.54, 122.27, 121.26, 119.13, 118.58, 111.18, 110.83, 109.66, 109.60, 55.06, 46.49, 32.47, 32.17, 29.20, 27.46, 21.08, 16.93, 14.24. *m*/*z* = 715.3 [M + H]^+^, 193.01, 305.14, 515.41, 350.11, 497.40, 365.18. MS [M + H]^+^ calculated for C_45_H_42_N_6_O_3_ = 715.33912; observed = 715.33650.

2-{[4′-(1,7′-dimethyl-2′-propyl-1H-[2,5′]bibenzoimidazolyl-3′-ylmethyl)-biphenyl-2-carbonyl]-amino}-3-(3H-imidazol-4-yl)-propionic acid (compound **2**):

White crystalline solid. Yield 48.1%. Melting point: 225–227 °C. IR (ATR) ν_max_ (cm^−1^): 743.31, 818.55, 1087.75, 1283.41, 1404.13, 1436.07, 1594.33, 2964.36, 3196.33. ^1^H NMR (400 MHz, DMSO) δ ppm: 6.63–7.47 (16H, m, ArH), 5.57 (2H, s, -CH_2_- at position C3′), 4.14 (1H, s, -CH from the amino acid side chain), 3.81 (3H, s, -CH_3_ at position N1), 2.89 (2H, s, -CH_2_-CH_2_-CH_3_), 2.62 (3H, s, -CH_3_ at position C7′), 1.79 (2H, m, -CH_2_-CH_2_-CH_3_), 0.97 (3H, t, *J* = 7.2 Hz, -CH_2_-CH_2_-CH_3_). ^13^C NMR (100 MHz, DMSO) δ ppm: 185.10, 168.33, 156.66, 154.51, 143.10, 142.92, 139.84, 139.03, 137.62, 137.11, 136.20, 135.21, 134.64, 130.35, 129.84, 129.17, 128.69, 128.21, 127.49, 126.84, 123.73, 123.68, 122.49, 122.23, 119.12, 110.85, 109.67, 54.39, 46.50, 33.71, 32.20, 29.19, 21.13, 16.92, 14.27. *m*/*z* = 651.3 [M − H]^−^, 303.08, 274.88, 259.82, 553.51, 273.79, 286.93. MS [M + H]^+^ calculated for C_39_H_37_N_7_O_3_ = 652.30306; observed = 652.30070.

2-{[4′-(1,7′-dimethyl-2′-propyl-1H-[2,5′]bibenzoimidazolyl-3′-ylmethyl)-biphenyl-2-carbonyl]-amino}-3-(4-hydroxy-phenyl)-propionic acid (compound **3**):

White crystalline solid. Yield 36.1%. Melting point: 238–240 °C. IR (ATR) ν_max_ (cm^−1^): 744.18, 827.95, 1090.62, 1246.26, 1440.18, 1514.31, 1593.43, 2922.39. ^1^H NMR (400 MHz, DMSO) δ ppm: 9.50 (1H, s, -COOH), 6.60–7.80 (18H, m, ArH), 5.54 (2H, s, -CH_2_- at position C3′), 4.20 (1H, s, -CH from the amino acid side chain), 3.81 (3H, s, -CH_3_ at position N1), 2.89 (2H, t, *J* = 7.6 Hz, -CH_2_-CH_2_-CH_3_), 3.00–3.02 and 2.78–2.82 (2H, m, -CH_2_ from the amino acid side chain), 2.63 (3H, s, -CH_3_ at position C7′), 1.80 (2H, m, -CH_2_-CH_2_-CH_3_), 0.98 (3H, t, *J* = 7.2 Hz, -CH_2_-CH_2_-CH_3_). ^13^C NMR (100 MHz, DMSO) δ ppm: 168.37, 156.66, 156.01, 154.51, 143.12, 142.80, 139.94, 139.04, 137.55, 137.06, 136.03, 135.21, 130.61, 130.39, 129.80, 129.72, 129.18, 128.70, 128.25, 127.39, 126.93, 123.65, 122.55, 122.29, 119.05, 115.09, 110.88, 109.67, 56.05, 46.59, 40.89, 36.81, 32.22, 29.21, 21.12, 16.92, 14.27. *m*/*z* = 678.3 [M + H]^+^, 305.09, 192.97, 497.22, 328.06, 515.24, 276.01. MS [M + H]^+^ calculated for C_42_H_39_N_5_O_4_ = 678.30748; observed = 678.30523.

2-{[4′-(1,7′-dimethyl-2′-propyl-1H-[2,5′]bibenzoimidazolyl-3′-ylmethyl)-biphenyl-2-carbonyl]-amino}-3-methyl-pentanoic acid (compound **4**):

White crystalline solid. Yield 43.9%. Melting point: 192–197 °C. IR (ATR) ν_max_ (cm^−1^): 744.01, 850.59, 1005.78, 1322.26, 1406.37, 1505.75, 1593.77, 2960.93, 3390.09. ^1^H NMR (400 MHz, DMSO) δ ppm: 7.11–7.57 (13H, m, ArH), 5.59 (2H, s, -CH_2_- at position C3′), 3,97 (1H, s, -CH from the amino acid side chain), 3.83 (3H, s, -CH_3_ at position N1), 2.90 (2H, m, -CH_2_-CH_2_-CH_3_), 2.63 (3H, s, -CH_3_ at position C7′), 2.54 (1H, s, -CH from the amino acid side chain), 1.83 (2H, m, -CH_2_-CH_2_-CH_3_), 1.00 (3H, t, *J* = 7.2 Hz, -CH_2_-CH_2_-CH_3_), 0.65 (3H, t, *J* = 6.8 Hz, -CH_3_ from the amino acid side chain), 0.53 (3H, d, *J* = 6.4 MHz, -CH_3_ from the amino acid side chain). ^13^C NMR (100 MHz, DMSO) δ ppm: 156.55, 154.47, 143.11, 142.94, 140.02, 138.97, 137.82, 137.10, 136.37, 135.17, 130.33, 129.73, 129.27, 128.67, 128.16, 127.54, 126.77, 123.77, 123.65, 122.49, 122.23, 119.12, 110.82, 109.64, 37.32, 32.21, 29.18, 25.19, 21.11, 16.92, 15.95, 14.31, 11.97. *m*/*z* = 626.4 [M − H]^−^, 303.12, 274.08, 287.07, 259.06, 260.07, 288.19. MS [M + H]^+^ calculated for C_39_H_41_N_5_O_3_ = 628.32822; observed = 628.32530.

{[4′-(1,7′-dimethyl-2′-propyl-1H-[2,5′]bibenzoimidazolyl-3′-ylmethyl)-biphenyl-2-carbonyl]-amino}-acetic acid (compound **5**):

White crystalline solid. Yield 37.4%. Melting point: 213–217.8 °C. IR (ATR) ν_max_ (cm^−1^): 744.34, 851.06, 1089.22, 1283.73, 1405.24, 1440.11, 1594.58, 2924.18, 3262.12. ^1^H NMR (400 MHz, DMSO) δ ppm: 7.12–7.64 (14H, m, ArH), 5.58 (2H, s, -CH_2_- at position C3′), 3.81 (3H, s, -CH_3_ at position N1), 2.89 (2H, s, -CH_2_-CH_2_-CH_3_), 2.61 (3H, s, -CH_3_ at position C7′), 1.78 (2H, m, -CH_2_-CH_2_-CH_3_), 0.97 (3H, s, -CH_2_-CH_2_-CH_3_). ^13^C NMR (100 MHz, DMSO) δ ppm: 169.02, 156.69, 154.47, 143.05, 142.89, 139.93, 139.03, 137.35, 137.09, 136.29, 135.19, 130.39, 129.92, 129.20, 128.71, 128.31, 127.54, 126.89, 123.75, 122.52, 122.25, 119.13, 110.85, 109.65, 46.50, 43.56, 32.19, 29.19, 21.15, 16.91, 14.27. *m*/*z* = 570.2 [M − H]^−^, 302.99, 274.04, 287.12, 259.26, 526.09, 315.06. MS [M + H]^+^ calculated for C_35_H_33_N_5_O_3_ = 572.26562; observed = 572.26328.

4-{[4′-(1,7′-dimethyl-2′-propyl-1H-[2,5′]bibenzoimidazolyl-3′-ylmethyl)-biphenyl-2-carbonyl]-amino}-butyric acid (compound **6**):

White crystalline solid. Yield 60.0%. Melting point: 175–177 °C. IR (ATR) ν_max_ (cm^−1^): 656.07, 744.00, 852.34, 1125.90, 1321.17, 1439.89, 1643.86, 2928.67, 3266.08. ^1^H NMR (400 MHz, DMSO) δ ppm: 8.20 (1H, s, -COOH), 7.12–7.78 (14H, m, ArH), 5.59 (2H, s, -CH_2_- at position C3′), 3.83 (3H, s, -CH_3_ at position N1), 3.00 (2H, d, *J* = 5.2 Hz, -NH-CH_2_-CH_2_-CH_2_-COOH), 2.88 (2H, t, *J* = 7.2 Hz, -CH_2_-CH2-CH_3_), 2.62 (3H, s, -CH_3_ at position C7′), 1.91 (2H, s, -NH-CH_2_-CH_2_-CH_2_-COOH), 1.79 (2H, m, -CH_2_-CH_2_-CH_3_), 1.47 (2H, s, -NH-CH_2_-CH_2_-CH_2_-COOH), 0.97 (3H, t, *J* = 6.8 Hz, -CH_2_-CH_2_-CH_3_). ^13^C NMR (100 MHz, DMSO) δ ppm: 169.38, 156.67, 154.50, 143.10, 142.84, 139.97, 138.91, 137.91, 137.06, 136.44, 135.27, 130.16, 129.61, 128.67, 128.06, 127.51, 126.75, 123.70, 123.65, 122.52, 122.26, 119.10, 110.84, 109.67, 46.47, 33.71, 32.20, 29.18, 25.21, 21.11, 16.91, 14.27. *m*/*z* = 598.2 [M − H]^−^, 303.09, 274.10, 259.14, 288.10, 566.40, 580.22. MS [M + H]^+^ calculated for C_37_H_37_N_5_O_3_ = 600.29692; observed = 600.29438.

2-{[4′-(1,7′-dimethyl-2′-propyl-1H-[2,5′]bibenzoimidazolyl-3′-ylmethyl)-biphenyl-2-carbonyl]-amino}-4-methyl-pentanoic acid (compound **7**):

White crystalline solid. Yield 34.0%. Melting point: 220–225 °C. IR (ATR) ν_max_ (cm^−1^): 744.16, 853.32, 1006.07, 1322.73, 1454.37, 1594.43, 2034.62, 2169.18, 2954.57, 3251.89. ^1^H NMR (400 MHz, DMSO) δ ppm: 7.73 (1H, s, -COOH), 7.10–7.64 (14H, m, ArH), 5.58 (2H, s, -CH_2_- at position C3′), 4.04 (1H, s, -CH from the amino acid side chain), 3.81 (3H, s, -CH_3_ at position N1), 2.90 (2H, t, *J* = 7.6 Hz, -CH_2_-CH_2_-CH_3_), 2.62 (3H, s, -CH_3_ at position C7′), 1.82 (2H, m, -CH_2_-CH_2_-CH_3_), 1,38–1.47 (3H, m, -CH_2_CH(CH_3_)_2_ from the amino acid side chain), 0.99 (3H, t, *J* = 7.6 Hz, -CH_2_-CH_2_-CH_3_), 0.69 (6H, m, 2 × -CH_3_ from the amino acid side chain). ^13^C NMR (100 MHz, DMSO) δ ppm: 168.67, 156.56, 154.48, 143.12, 142.95, 140.01, 138.97, 137.81, 137.12, 136.29, 135.19, 130.29, 129.71, 128.69, 128.24, 127.47, 126.72, 123.78, 123.66, 122.47, 122.21, 119.13, 110.81, 109.64. *m*/*z* = 626.1 [M − H]^−^, 302.97, 273.88, 286.96, 258.90, 260.09, 288.11. MS [M + H]^+^ calculated for C_39_H_41_N_5_O_3_ = 628.32822; observed = 628.32598.

2-{[4′-(1,7′-dimethyl-2′-propyl-1H-[2,5′]bibenzoimidazolyl-3′-ylmethyl)-biphenyl-2-carbonyl]-amino}-3-phenyl-propionic acid (compound **8**):

White crystalline solid. Yield 42.3%. Melting point: 198–202 °C. IR (ATR) ν_max_ (cm^−1^): 700.82, 743.81, 851.49, 1005.88, 1322.44, 1405.04, 1453.89, 1594.56, 2164.96, 2925.48, 3283.72. ^1^H NMR (400 MHz, DMSO) δ ppm: 7.99 (1H, m, -COOH), 6.86–7.75 (19H, m, ArH), 5.56 (2H, s, -CH_2_- at position C3′), 4.28 (1H, s, -CH from the amino acid side chain), 3.81 (3H, s, -CH_3_ at position N1), 3.13 (1H, m, 1 × -H from the -CH_2_ in the amino acid side chain), 2.86 (3H, m, -CH_2_-CH_2_-CH_3_ and 1 × -H from the -CH_2_ in the amino acid side chain), 2.66 (3H, s, -CH_3_ at position C7′), 1.79 (2H, m, -CH_2_-CH_2_-CH_3_), 0.97 (3H, t, *J* = 7.2 Hz, -CH_2_-CH_2_-CH_3_). ^13^C NMR (100 MHz, DMSO) δ ppm: 168.57, 156.62, 154.52, 143.13, 142.97, 139.88, 139.69, 138.94, 137.52, 137.12, 136.04, 135.29, 130.30, 129.80, 129.62, 128.98, 128.71, 128.34, 128.12, 127.34, 126.58, 126.04, 123.81, 123.67, 122.48, 122.22, 119.15, 110.82, 109.69, 55.82, 46.38, 40.87, 37.61, 32.20, 29.18, 21.08, 16.94, 14.25. *m*/*z* = 662.3 [M + H]^+^, 305.06, 312.05, 192.96, 515.20, 497.27, 275.99. MS [M + H]^+^ calculated for C_42_H_39_N_5_O_3_ = 662.31257; observed = 662.30963.

### 4.3. Cell Lines and Viability Assay

A375, MRC-5, and HaCaT cell lines were obtained from the American Type Culture Collection (ATCC). 518A2 cell line was kindly provided by Dr. Milica Pešić. All cell lines were cultured in RPMI 1640 medium supplemented with 2 g/L glucose, 10% fetal bovine serum (FBS), and antibiotics. The A375R cell line was established in our laboratory according to the procedure previously described [5] and was maintained in RPMI containing 2 µM vemurafenib (Genentech, Inc., South San Francisco, CA, USA). Cell viability was assessed using the MTT assay (Sigma Aldrich, #M5655). Briefly, A375, 518A2, and A375R cells were seeded in 96-well plates at a density of 3000 cells/well, while MRC-5 and HaCaT cells were seeded at 5000 and 7000 cells/well, respectively. Cells were treated with five different concentrations of each of the eight tested compounds and telmisartan (DMSO did not exceed 0.6% in the final treatment solutions). In the experimental setting investigating the effects of amino acids and telmisartan, cells were treated with combination of 7.5 µM N-methyltryptophan, 6.25 µM phenylalanine, 6.75 µM tyrosine, and 18, 19, or 19.5 µM telmisartan, corresponding to the molar ratios of amino acid and telmisartan as structural components in 25 µM of compounds **1**, **3**, and **8**, respectively. After 72 h of incubation, absorbance was measured at 570 nm using a Multiskan SkyHigh microplate reader (Thermo Fisher Scientific, Helsinki, Finland). Values were corrected by subtracting the absorbance measured in wells containing cell-free medium with tested compounds. The IC_50_ value was determined as the concentration of compound required to reduce 3-(4,5-dimethylthiazol-2-yl)-2,5-diphenyltetrazolium bromide (reduction by 50% compared with control samples treated with DMSO.

### 4.4. Subcellular Fractionation

Stepwise separation and preparation of cytoplasmic, membrane, nuclear soluble, chromatin-bound, and cytoskeletal protein extracts from A275 cells was performed with Subcellular Protein Fractionation Kit (#78840, Thermo Scientific), according to the manufacturer’s instructions. 1 × 10^6^ A375 cells per condition were treated with 25 μM telmisartan or compounds **1**, **3**, and **8** for 24 h. Cells were then trypsinized, washed with PBS and pelleted. For whole cell lysates, cell pellet was dissolved in RIPA buffer on ice. Fractions and whole cell lysates were stored at −80 °C until use. Protein concentration of lysates was determined with Pierce BCA Protein assay kit (Thermo Fisher #23225), and cell accumulation was calculated as μmol of compound per μg of cell protein and normalized to telmisartan. Concentrations of telmisartan and its derivatives were determined using liquid chromatography-tandem mass spectrometry (LC-MS/MS) method in positive HESI mode. The analysis was performed on UHPLC chromatograph ACELLA (Thermo Fisher Scientific Inc., Madison, WI, USA), coupled to a triple quadrupole mass spectrometer TSQ Quantum Access MAX (Thermo Fisher Scientific Inc., Madison, WI, USA) with heated electrospray ionization (HESI) interface. The column was Zorbax Extend C18 (150 mm × 4.6 mm, 5 µm particle size). Mobile phase was acetonitrile/0.1% formic acid = 60:40 (*v*/*v*), flow rate was 0.5 mL min^−1^, column temperature was set to 25 °C, and the injection volume was 10 µL. Experiment was repeated twice.

### 4.5. Ca^2+^ Staining in Live Cells

Fluo-4 AM calcium indicator dye (F14201, Molecular Probes, Eugene, OR, USA) was used to observe Ca^2+^ dynamics upon treatment, per manufacturer’s instructions. Briefly, 5000 A375 cells/well were seeded in 96-well optical bottom black cell culture plates (165305, Thermo Scientific). Fluo-4 AM stock solution in DMSO was mixed with equal volume of 20% Pluronic F-127 to assist in dispersion of AM esters before dilution into the loading medium. Twenty-four hours after seeding medium was removed, and cells were washed with HBBS without calcium and magnesium. Cells were stained in HBSS medium with 1.25 μM Fluo-4-AM for 30 min at 37 °C. After incubation, cells were washed and incubated in complete medium (10%FBS RPMI) to allow de-esterification for 30 min and then treated with 1 μM human Angiotensin II (A9525, Sigma Aldrich), 50 μM telmisartan, compounds **1**, **3**, and **8**, or the combinations. Images were taken before addition of the treatment (0 min) and after 2 min of treatment on Zeiss PALM MicroBeam Axio Observer Z1 microscope (Carl Zeiss MicroImaging GmbH, Oberkochen, Germany) with Zeiss LD Plan-Neofluar 20×/0.4 Korr, ∞/0–1.5 mm lens and AxioVision SE64 Rel.4.9.1 software. Experiment was repeated three times. Integrated density of Fluo-4 fluorescence at 2 min of exposure to treatment was calculated with Image J, and normalized to the baseline fluorescence at time 0, in at least two random fields for each condition. Conditions were compared with one-way ANOVA and Tukey’s multiple comparisons test. *p* < 0.05 was considered statistically significant.

### 4.6. Cell Cycle Distribution Analysis

A375 and A375R cells were seeded in 6-well plates at a density of 150,000 cells per well. On the following day, the cells were treated with 25 µM concentrations of compounds **1**, **3**, and **8**, as well as telmisartan. After 72 h of incubation, the cells were harvested using trypsin, washed with phosphate-buffered saline (PBS), fixed in ethanol, and stored at −20 °C until analysis. Ethanol-fixed cells were treated with RNase A (1 mg/mL) and stained with propidium iodide (PI; 400 µg/mL). A total of 20,000 events were recorded per sample. Quantitative analysis of the cell cycle phase distribution was performed by flow cytometry using a FACSCalibur flow cytometer (Becton Dickinson, San Jose, CA, USA) and BD CellQuest Pro Version 5.2.1 software. Experiment was performed three times. Cells under treatment were compared to controls for each cell phase separately with one-way ANOVA and Dunnett’s multiple comparison tests. *p* < 0.05 was considered statistically significant.

### 4.7. Mitochondrial Morphology Staining

For imaging of the mitochondrial morphology in live cells, A375 cells seeded in 96-well optical bottom plates, as above, were treated for 24 h with 25 μM telmisartan, compound **1**, **3**, or **8**. Treatment was washed away, and cells were stained with 500 nM MitoTracker Green FM (#9074, Cell signaling Technology, Danvers, MA, USA) in RPMI without serum for 30 min at 37 °C. Cells were imaged on Zeiss PALM MicroBeam Axio Observer Z1 microscope (Carl Zeiss MicroImaging GmbH, Oberkochen, Germany) with Zeiss LD Plan-Neofluar 20×/0.4 Korr, ∞/0–1.5 mm lens and AxioVision software. Mitochondrial shape was quantified in images from two biological experimental repeats, in three random fields for each condition, using Image J Analyze Particles function. Images were first processed to 8-bit binary, and circularity and aspect ratio (major axis/minor axis) were calculated and plotted with Prism 9 (GraphPad Software, LLC, Boston, MA, USA). Treatments were compared with untreated control using one-way ANOVA and Dunnett’s multiple comparisons test. *p* < 0.05 was considered statistically significant.

### 4.8. Measurement of the Mitochondrial Potential

Changes in mitochondrial membrane potential were analyzed using the JC-1 Mitochondrial Membrane Potential Assay Kit (ab113850) according to the manufacturer’s instructions. Briefly, A375 cells were treated with telmisartan and its derivatives (compounds **1**, **3**, and **8**). After 24 h, cells were incubated with the JC-1 staining solution at 37 °C for 30 min in dark, washed with 1X Dilution Buffer, and analyzed by flow cytometry. FCCP (carbonyl cyanide 4-(trifluoromethoxy) phenylhydrazone) was used as a positive control to induce mitochondrial depolarization. The ratio of red J-aggregates to green JC-1 monomers was calculated to assess changes in mitochondrial membrane potential. Experiment was performed twice.

### 4.9. Stability in Biological Media

To test stability in biological media, three best drug candidates (compounds **1**, **3**, and **8**) were dissolved in 0.05 M hydrochloric acid, pH 2.0, and phosphate buffer, pH 5.5 (prepared by dissolving sodium dihydrogen phosphate dihydrate and disodium hydrogen phosphate in distilled water, followed by the adjustment of pH by ortho-phosphoric acid) and RPMI 1640 medium. Stock solutions in DMSO (2 mg/mL) were diluted by corresponding medium to obtain working solutions (0.02 mg/mL) and incubated at room temperature in 0.05 M hydrochloric acid (2 h), phosphate buffer pH 5.5 (5 h), and RPMI 1640 medium (72 h). Compounds were injected freshly prepared, and after incubation time, the differences in concentrations was expressed as percentages. Quantification was performed on Dionex Ultimate 3000 HPLC system (Thermo Fisher Scientific, Dreieich, Germany), consisting of Dionex Ultimate 3000 quaternary pump, autosampler, and DAD detector. The column was Zorbax Extend C18 (150 mm × 4.6 mm, 5 µm particle size). The mobile phase was 0.1% formic acid/acetonitrile = 50/50 (compound **1**) and 60/40 (compounds **3** and **8**). The column temperature was adjusted to 25 °C, and the flow rate was 0.5 mL/min. The detection was performed at 300 nm.

### 4.10. Statistical Analysis

All results were expressed as mean ± SD, unless otherwise stated. Statistical analysis was performed using Prism 9 (GraphPad Software, LLC). Differences between control and treatments were analyzed with one-way ANOVA, with a *p* < 0.05 considered as statistically significant, with Tukey’s or Dunnett’s multiple comparison tests. Statistical analysis was performed only in experiments performed in at least three biological replicates.

## 5. Conclusions

In this study, a series of novel telmisartan–amino acid conjugates aimed at enhancing anticancer efficacy while minimizing antihypertensive activity was designed and synthesized. Among the synthesized compounds, three telmisartan conjugates (with N-methyl-L-tryptophan—compound **1**, L-tyrosine—compound **3**, and L-phenylalanine compound **8**) showed significantly improved cytotoxic effects in BRAF V600E-mutated cell lines A375 and 518A2 (at least twofold higher activity than telmisartan). In addition, compound **3** demonstrated more than threefold higher activity against vemurafenib-resistant melanoma cells (A375R), with increased selectivity over non-malignant cells when compared to telmisartan. Their increased activity could be attributed to enhanced cellular uptake and mitochondrial disruption, leading to cell cycle arrest and apoptosis. Molecular docking analysis and calcium signaling assays suggest that compounds **1**, **3**, and **8** exhibited reduced interaction with AT1 receptors, which should result in the diminishing of antihypertensive activity. The results obtained in this study indicate that compounds **1**, **3**, and **8** retain and even surpass the anticancer properties of telmisartan, while alleviating its antihypertensive effects. Therefore, these derivatives represent promising candidates for further preclinical development as antimelanoma chemotherapeutics, especially in patients without hypertension and resistant to current BRAF-targeted strategies.

## Figures and Tables

**Table 1:** Docking scores of designed compounds and telmisartan.

Compound	Score
Telmisartan	−14.13
Telmisartan–glycine	−12.54
Telmisartan–L-alanine	−11.94
Telmisartan–L-valine	−10.89
Telmisartan–L-leucine	−8.87
Telmisartan–L-isoleucine	−8.95
Telmisartan–gamma-aminobutyric acid	−10.97
Telmisartan–beta-alanine	−12.21
Telmisartan–L-glutamic acid	−9.82
Telmisartan–L-proline	−11.99
Telmisartan–L-tyrosine	−8.10
Telmisartan–L-phenylalanine	−8.52
Telmisartan–N-methyl-L-tryptophan	−4.14
Telmisartan–L-tryptophan	−4.73
Telmisartan–L-histidine	−8.73
Telmisartan–L-taurine	−11.08

**Table 2:** Cell viability was assessed using the MTT assay following 72 h of treatment. Compounds were tested at concentrations ranging from 9.4 to 150 µM. The IC_50_ values are expressed as the mean ± standard deviation derived from three independent experiments.

Compounds	IC_50_ [µM]	
	A375	518A2
**1**	27.21 ± 0.63	23.47 ± 2.08
**2**	>150	>150
**3**	32.07 ± 2.38	30.99 ± 2.38
**4**	42.95 ± 6.02	48.99 ± 3.17
**5**	86.22 ± 0.64	81.51 ± 6.28
**6**	116.03 ± 6.94	89.21 ± 7.37
**7**	43.21 ± 3.55	39.74 ± 2.40
**8**	27.22 ± 0.58	28.35 ± 2.66
**Telmisartan**	59.05 ± 4.17	70.21 ± 3.83

**Table 3:** IC_50_ values of tested compounds and telmisartan in A375R, HaCaT, and MRC-5 cells. Cell viability was determined by MTT assay after 72 h of treatment. IC_50_ values are reported as mean ± standard deviation from three independent experiments.

Compounds	IC_50_ [µM]		
	A375R	HaCaT	MRC-5
**1**	19.67 ± 8.87	43.92 ± 3.42	26.66 ± 2.48
**3**	21.08 ± 6.31	63.46 ± 0.23	50.74 ± 0.28
**8**	8.84 ± 1.24	70.75 ± 6.99	31.54 ± 5.87
**Telmisartan**	29.23 ± 3.88	147.96 ± 2.89	70.35 ± 5.61

**Table 4:** Compound cell accumulation and distribution normalized to telmisartan from two biological replicates.

Compounds		Cellular Fractions				
	Whole Lysate	Cytoplasm	Membrane	Nucleus Soluble	Chromatin Bound	Cytoskeleton
**1**	12.08	17.80	106.59	30.95	36.22	21.14
**3**	4.57	3.64	4.62	0.85	0.52	0.69
**8**	4.41	11.17	274.15	13.79	11.90	7.44
**Telmisartan**	1.00	1.00	1.00	1.00	1.00	1.00

## Data Availability

The original contributions presented in this study are included in the article. Further inquiries can be directed to the corresponding authors.
